# Identification of additional regulatory RNPs that impact rRNA and U6 snRNA methylation

**DOI:** 10.1242/bio.036095

**Published:** 2018-07-23

**Authors:** Marilyn F. Burke, Madelyn K. Logan, Michael D. Hebert

**Affiliations:** Department of Cell and Molecular Biology, The University of Mississippi Medical Center, Jackson, MS 39216-4505, USA

**Keywords:** Cajal body, Nucleolus, snoRNPs

## Abstract

Ribosomes can be heterogeneous, and the major contributor to ribosome heterogeneity is variation in rRNA modification. There are two major types of rRNA modification, pseudouridylation and ribose methylation. In humans, the majority of these rRNA modifications are conducted by two classes of small nucleolar ribonucleoproteins (snoRNPs), which contain a guide RNA (small nucleolar RNA, snoRNA) complexed with proteins. Box H/ACA snoRNPs conduct pseudouridylation modifications and box C/D snoRNPs generate ribose methylation modifications. It is unclear how ribosome heterogeneity is accomplished in regards to the understanding of the signals and factors that regulate rRNA modifications. We have recently reported that a new class of RNP, that we term regulatory RNP (regRNP), may contribute to rRNA modification as well as the modification of nucleolar trafficked U6 snRNA, via interactions with snoRNPs. Here we report the identification of additional regRNP activities that influence the methylation of two sites within 18S rRNA, two sites within 28S rRNA and one site within U6 snRNA. These findings provide additional proof that regulation of snoRNP activity contributes to ribosome heterogeneity.

## Abstract (precis)

**Summary:** The major contributor to ribosome heterogeneity is variation in rRNA modification by methylation and pseudouridylation. Our work provides additional evidence for the regulation of snoRNP activity by regRNPs, which alters levels of methylation.

## INTRODUCTION

An exciting emerging concept is that of ribosome heterogeneity leading to specialized ribosomes. This concept is based on the realization that ribosomes are not all the same, all the time, but can vary in response to different physiological or pathological situations (Lafontaine, 2015). By having specialized ribosomes, the cell is able to increase or decrease the translation of certain mRNAs resulting in a protein composition that is optimized for a given environment. In pathological situations specialized ribosomes may contribute to, or perpetuate, the disease state at the level of translation by decreasing the amount of disease-preventing proteins and/or increasing the amount of disease-contributing proteins. The importance of ribosome heterogeneity leading to specialized ribosomes has recently been demonstrated as a major contributor to tumorigenesis (Marcel et al., 2015, 2013; Truitt and Ruggero, 2016). These studies found that the reduction of p53 (a common mutation in cancer cells) results in specialized ribosomes with a lower fidelity (i.e. stop codons are bypassed) and a greater likelihood to initiate translation through internal ribosome entry sequences (IRESs) (Belin et al., 2009; Marcel et al., 2015, 2013). Consequently, reduction of p53 results in the increased translation of IRES-containing messages whose products, such as IGF-1R, c-myc, VEGF-A and FGF1, promote tumor development (Marcel et al., 2015).

There are many contributors to the generation of ribosome heterogeneity, including variation in the ribosomal protein complement and heterogeneity of translation factors (Lafontaine, 2015). However, the chief contributor to ribosome heterogeneity is ribosomal RNA (rRNA) modification (Lafontaine, 2015). There are two major kinds of rRNA modification: 2′-*O*-ribose methylation and pseudouridylation. In humans, most of these modifications are conducted by a class of ribonucleoproteins (RNPs) known as small nucleolar RNPs (snoRNPs). As their name implies, snoRNPs are comprised of protein and RNA components. The RNA component of the snoRNP (the snoRNA guide) base pairs with a target region within rRNA, which then facilitates the modification of the target by the enzymatic (protein) component of the snoRNP. There are approximately 200 modifications in human rRNA and, considering that each site of modification is governed by a specific snoRNP, alterations in the activity of these snoRNPs are likely to be the greatest source of ribosome heterogeneity (Lafontaine, 2015).

There are two classes of snoRNPs: box C/D and box H/ACA. Box C/D snoRNPs contain fibrillarin (which conducts ribose methylation) and box H/ACA snoRNPs contain dyskerin (which conducts pseudouridylation) (Massenet et al., 2017). The two classes of snoRNPs are defined by their small nucleolar RNA (snoRNA) component, which contains consensus C/D (C=RUGAUGA, D=CUGA) or box H/ACA (H=ANANNA, ACA=ACA) sequence elements. As mentioned above, the reduction of p53 found in many cancers results in the alteration of the ribosome, which in turn increases the level of proteins promoting tumorigenesis (Marcel et al., 2015, 2013; Truitt and Ruggero, 2016). This change in ribosome heterogeneity in normal cells versus cancer cells is a consequence of p53 directly regulating fibrillarin levels. In non-transformed cells, p53 down regulates fibrillarin levels. However, in cancer cells lacking functional p53 the level of rRNA methylation is increased because fibrillarin levels are increased. This results in greater amounts of box C/D snoRNPs (Marcel et al., 2013). The increase in rRNA methylation, therefore, is a direct result of increased snoRNP activity and is the basis for the increased translation of mRNAs that produce tumorigenesis-inducing proteins (Marcel et al., 2015).

In addition to the nucleolus, the nucleus contains numerous other domains, territories and bodies. Like the nucleolus, the number, composition and activity of these structures can change in response to developmental or environmental cues or disease state. One of these structures is the Cajal body (CB). The CB participates in the formation of many different types of RNPs (Kiss, 2004), one of which is the small nuclear RNP (snRNP) which plays a vital role in pre-mRNA splicing. In addition to snRNPs, the CB also takes part in the biogenesis of small CB-specific RNPs (scaRNPs) and snoRNPs. scaRNPs conduct modifications (ribose methylation and pseudouridylation) of the snRNA component of snRNPs. These snRNP modifications are crucial for spliceosomal function. There are three different classes of scaRNPs defined by conserved motifs present in the scaRNA: box C/D, box H/ACA and mixed domain. These different types of scaRNAs base pair with target snRNA and guide the activity of enzymes present in the scaRNP complex to modify specific sites within the snRNA. As mentioned above, snoRNPs are responsible for the modification of rRNA, which is important for ribosomal activity and the major contributor towards ribosome heterogeneity. Some scaRNAs (scaRNA 2, 9 and 17) can be processed into smaller RNA fragments that accumulate in the nucleolus (Tycowski et al., 2004). For example, scaRNA2 can be processed to generate a fragment known as mgU2-61, which is predicted to serve as a methylation guide (mg) for U2 snRNA at position 61 (hence mgU2-61). Interestingly however, U2 snRNA does not have a clear nucleolar step in its biogenesis pathway. Indeed, when considering all the major spliceosomal snRNAs (U1, U2, U4, U5 and U6), only the RNA polymerase III-derived U6 snRNA has a clear nucleolar biogenesis step (Kiss, 2004). The function of these nucleolar-enriched fragments derived from scaRNA 2, 9 and 17 is, therefore, not clear, but we have recently reported the possibility that they may form regulatory RNPs (regRNPs) that regulate rRNA modification by influencing snoRNP activity (Poole et al., 2017). Specifically, we have provided evidence that the scaRNA17 processed nucleolar fragment can interact – via a single-stranded loop region – with the snord16 snoRNP (Poole et al., 2017). Methylation of A484 in 18S rRNA is mediated by snord16. We hypothesized that the interaction of the scaRNA17 fragment (regRNP17) with snord16 could disrupt the interaction of snord16 with rRNA, and thereby impact the methylation of 18S A484. This hypothesis is supported by methylation analysis of 18S rRNA A484, which showed an increase in A484 methylation upon reduction of scaRNA17, suggesting that regRNP17 negatively regulates A484 methylation (Poole et al., 2017).

Here, we examine additional regRNP activities derived from scaRNA17 and scaRNA2. We have also identified snord111B as having regRNP functions. Collectively, our work expands the known sites that can be altered by regRNPs to four in rRNA and one in U6 snRNA. By investigating other RNA interactions to a conserved loop motif found in many snoRNAs, it is likely that additional regRNP activities will be identified. The identification of other regRNPs will further clarify how ribosomal heterogeneity is accomplished.

## RESULTS

**Fig. 1.**
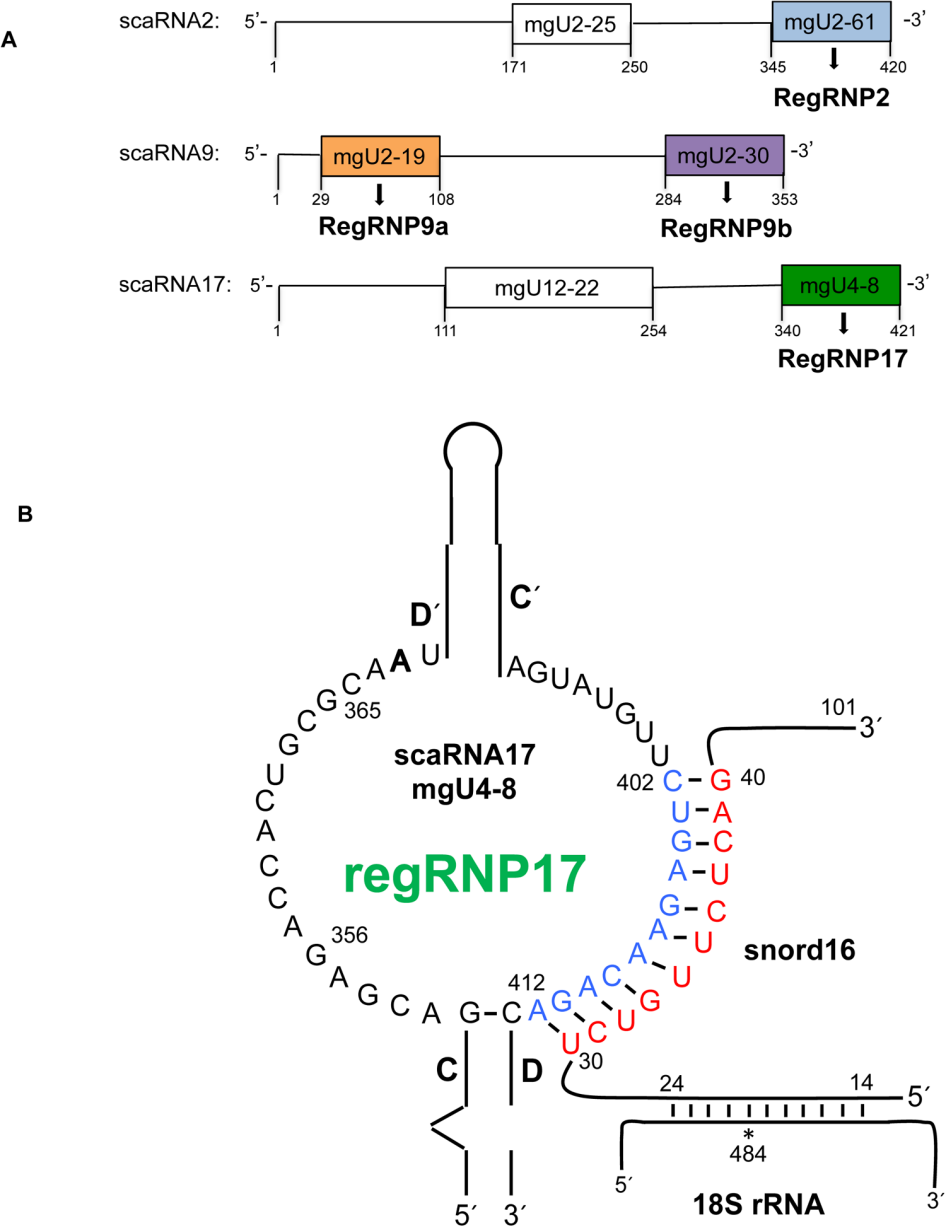
**Regulatory RNPs derived from scaRNA 2, 9 and 17.** (A) Schematic representation of scaRNA 2, 9 and 17. The colored boxed regions indicate processed fragments derived from the full-length scaRNA. These fragments accumulate in the nucleolus where we hypothesize they form regulatory RNPs (regRNPs). ScaRNA 2 and 17 are encoded by independently transcribed genes, whereas scaRNA9 is encoded in the intron of a host gene. (B) Predicted secondary structure of the regRNP17 fragment derived from scaRNA17 [adapted from Tycowski et al. (2004)]. A potential interaction of regRNP17 with snord16 is shown.

There are 29 known scaRNAs in humans (Jorjani et al., 2016), but only scaRNA 2, 9 and 17 have been reported to have additional internal processing that releases stable mg fragments that accumulate in the nucleolus (Tycowski et al., 2004). We hypothesize that these nucleolar enriched fragments form regulatory RNPs (regRNPs) that impact rRNA modification by interacting with snoRNPs (Poole et al., 2017). Specifically, scaRNA2 is processed to release mgU2-61 (regRNP2), scaRNA9 releases mgU2-19 (regRNP9a) and mgU2-30 (regRNP9b), and scaRNA17 releases mgU4-8 (regRNP17) (Fig. 1A). ScaRNA2 and scaRNA17 are generated from independently transcribed genes, while scaRNA9 is derived from the intronic region of the *CEP295* host gene. We have previously reported that the scaRNA17 fragment mgU4-8, regRNP17, has a negative regulatory effect on the 18S rRNA 484 methylation site (Poole et al., 2017). The guide RNA for the methylation of 18S rRNA at position 484 is snord16. An alignment of the antisense loop of regRNP17 with snord16 is shown in Fig. 1B, and similar interactions are observed between the antisense loops of regRNP2, regRNP9a and regRNP9b with other snoRNAs (Poole et al., 2017).

### 18S rRNA A484 methylation is altered upon snord16 or scaRNA17 reduction

**Fig. 2.**
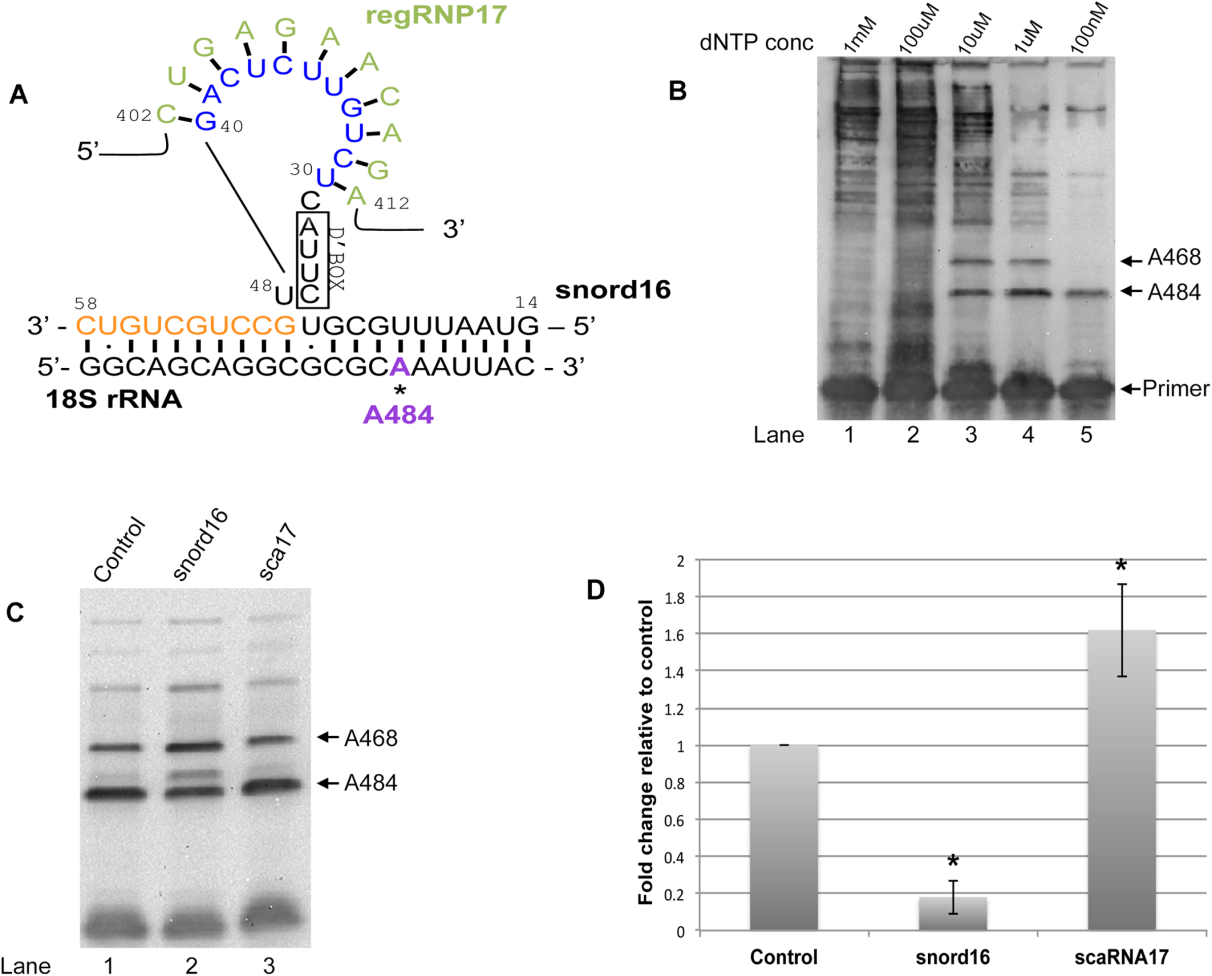
**ASO-mediated differential methylation of 18S rRNA A484.** (A) Schematic representation of the interaction of regRNP17 with snord16. The guide RNA for the methylation of A484 within 18S rRNA is snord16. Extra base pairings of snord16 with 18S rRNA, described by van Nues et al. (2011), are indicated by orange nucleotides. These extra base pairings of snord16 with 18S rRNA form a loop in snord16, and this snord16 loop contains the regRNP17 interacting region (blue nucleotides). (B) Primer extension assay to detect methylated sites within RNA. A digoxigenin labeled primer designed to interrogate 18S rRNA A484 and A468 was used in reactions with reverse transcriptase and RNA. Decreasing dNTP concentrations (indicated) were used for each reaction. The location of the primer and methylation stops for A484 and A468 are indicated. (C) ASO-mediated alteration of A484 methylation. Cells were treated for 48 h with control, snord16 or scaRNA17 ASOs. Isolated RNA was subjected to primer extension with low (2.5 uM) levels of dNTP. The location of the A484 and A468 methylation sites are shown. Quantification of this and other data are shown in D. For this quantification the signal for A484 was divided by the A468 signal and this ratio was normalized to that obtained with control ASO. Treatment with snord16 ASO results in an 82% decrease in the methylation of A484 (*n*=3) and treatment with scaRNA17 ASO results in a 1.6-fold increase in the methylation of A484 (*n*=3), compared to control. Asterisks indicate *P*<0.05.

We have previously reported that the reduction of scaRNA17 by RNAi increases the amount of 18S rRNA A484 methylation, suggesting that regRNP17 serves as a negative regulator of A484 methylation (Poole et al., 2017). The interaction of regRNP17 with snord16 takes place within a loop of snord16 that is formed by ‘extra base pairings’ of snord16 with 18S rRNA (Fig. 2A). Methylation of rRNA by some box C/D snoRNPs is facilitated by ‘extra base pairings’ between the snoRNA and target rRNA (van Nues et al., 2011). Most of these extra base pairings are the result of loops within the snoRNA, allowing for additional snoRNA–rRNA interactions. The interaction between snord16 with 18S rRNA is an example of an association that contains extra base pairings (Fig. 2A). An additional ten base pairings (orange colored nucleotides in Fig. 2A) between snord16 and 18S rRNA is made possible by a loop of snord16 between nucleotide 24 and 48. By having this arrangement of interactions it is expected that the methylation of A484 of 18S rRNA is increased as a consequence of these extra base pairings compared to the level of methylation if only nucleotides 14-24 of snord16 base paired with 18S rRNA. We previously found evidence that the interaction of regRNP17 with the loop region of snord16 disrupts base pairing between snord16 and 18S rRNA, resulting in a decrease in the level of 18S rRNA A484 methylation (Poole et al., 2017).

To follow up on these observations we examined 18S rRNA A484 methylation in cells treated with antisense oligonucleotides (ASOs) to scaRNA17 and snord16. RNA isolated from cells treated for 48 h with ASOs was subjected to primer extension with low levels of dNTPs. Low levels of dNTP cause reverse transcriptase to pause near sites of ribose methylation (Maden et al., 1995). Fig. 2B is a representative primer extension assay with decreasing levels of dNTP. The higher 1 mM and 100 µM dTNP concentrations do not produce a stop while the 10 µM, 1 µM and 100 nM dNTP concentrations reveal the A484 methylation site. In addition to the A484 site, the A468 methylation site of 18S rRNA can also be observed when using 10 µM and 1 µM dNTP in the reverse transcriptase reaction. As determined using primer extension with low dNTP shown in Fig. 2C, the amount of A484 methylation in cells treated with snord16 ASO (lane 2) is reduced compared to that observed in cells treated with control ASO (lane 1). In contrast, the amount of A484 methylation is increased in cells treated with scaRNA17 ASO (lane 3). To quantify this data we divided the A484 signal by the A468 signal for each condition, and then normalized these values by that obtained with control ASO (Fig. 2D). With the control condition set to 100% it can be observed that, as expected, decreased snord16 levels significantly reduce the relative amount of A484 methylation by 80%. However, reduction of scaRNA17 by ASO significantly increases the relative amount of A484 methylation by 1.6-fold. The level of snord16 and scaRNA17 in these experiments was typically reduced by 70% and 60%, respectively, as determined by qRT-PCR. These findings demonstrate that methylation of the A484 site of 18S rRNA is subjected to negative regulation imparted by scaRNA17, possibly via the nucleolus-enriched regRNP17.

### Nucleolar trafficked U6 snRNA methylation is altered upon scaRNA17 reduction

**Fig. 3.**
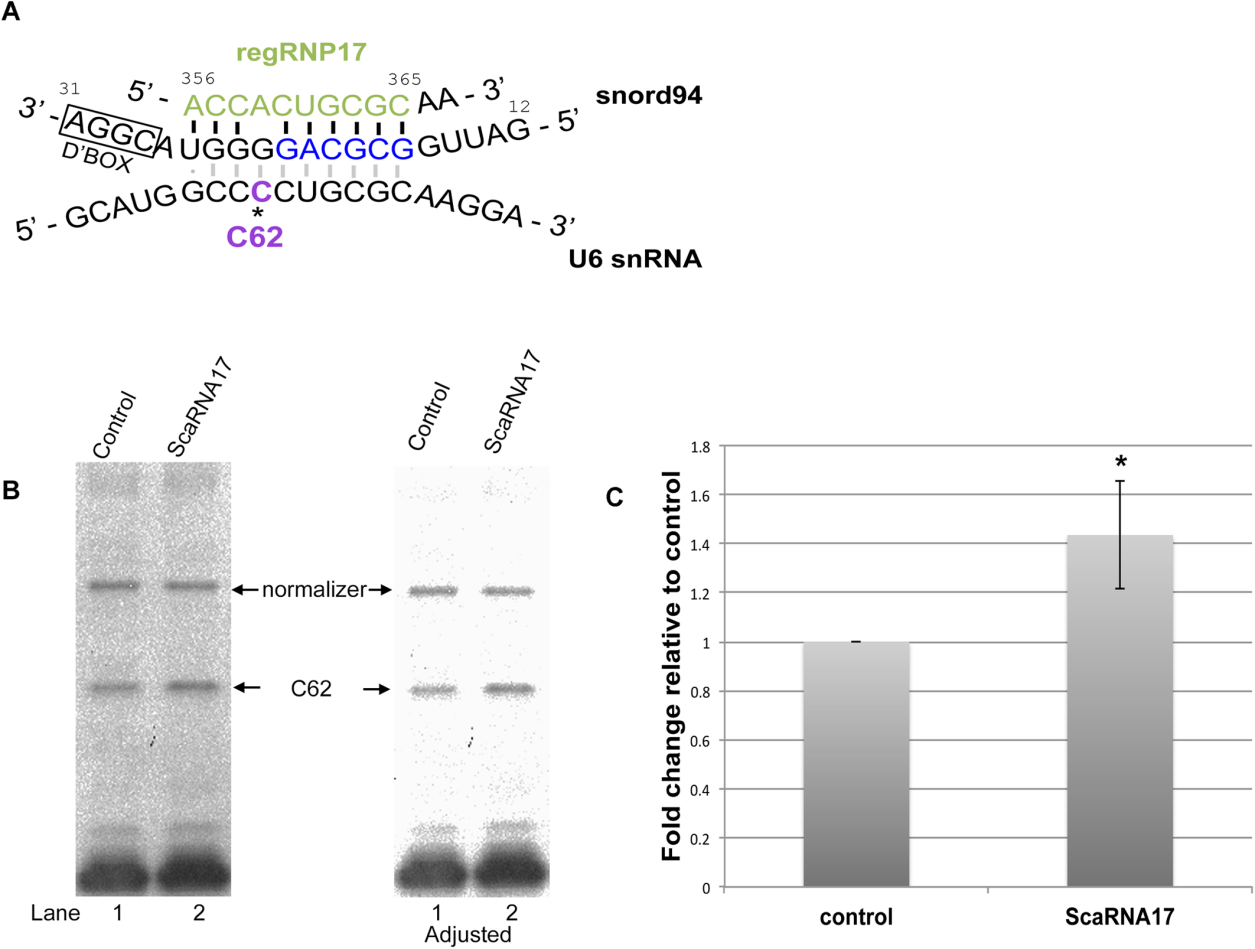
**Methylation of U6 snRNA at site C62 is increased when scaRNA17 is reduced.** (A) Schematic of the complimentary alignment of the guide snoRNA (snord94) with U6 snRNA that directs methylation of C62. The alignment of scaRNA17-derived regRNP17 with snord94 is shown, and it is predicted that the interaction of regRNP17 with snord94 will decrease the interaction of snord94 with U6 snRNA (denoted by light gray interactions). Note that regRNP17 nucleotides CUGCGCAA are also found in U6 snRNA. (B) RNA isolated from cells treated with control or scaRNA17 siRNA was subjected to primer extension in the presence of low (5 uM) dNTP levels. The location of the primer and methylation stop for C62 is indicated. A representative result is shown. The right panel is an adjusted image of the left panel, in order to more easily visualize the differences between C62 methylation in control versus scaRNA17 knockdown. (C) Quantification of the data in B, along with other results, was used to generate a histogram showing that C62 methylation increases 1.4-fold in cells with scaRNA17 knockdown compared to control (a normalizer band was used as an internal control, *n*=4, *P=0.008).

To expand our understanding of the role scaRNA17/regRNP17 may play in regulating methylation in the nucleolus, we next examined the interaction of regRNP17 with snord94. Snord94 is the predicted mg for C62 of the U6 snRNA (Fig. 3A). U6 snRNA is the only major spliceosomal snRNA that has a clear nucleolar biogenesis pathway (Kiss, 2004) and thus could be subjected to regulation by the nucleolus-enriched scaRNA fragments derived from scaRNA 2, 9 and 17. BLAST searches demonstrate that the antisense loop of regRNP17 can align with snord94 (Fig. 3A). Note that the alignment of regRNP17 with snord94 comprises nucleotide 356-365 of scaRNA17 and is on the 5′ side of the loop compared to the 3′ side of the antisense loop (nucleotide 402-412) which aligns with snord16 (Figs 1B and 2A). Eight nucleotides of the regRNP17 5′ loop (CUGCGCAA) that align with snord94 are exactly conserved in U6 snRNA and this sequence is in the region of U6 snRNA that interacts with snord94 (Fig. 3A). Based on the alignment of regRNP17 with snord94, we predicted that the interaction of regRNP17 with snord94 would disrupt the interaction of snord94 with U6 snRNA (denoted by faint bands between snord94 and U6 snRNA in Fig. 3A). If true, we would predict that regRNP17 would act as a negative regulator of C62 methylation in U6 snRNA. To test this hypothesis we transfected HeLa cells with control or scaRNA17 ASOs for 48 h and subjected the isolated RNA to primer extension analysis with low levels of dNTP to analyze C62 methylation. Fig. 3B is a representative primer extension assay indicating that a reduction in scaRNA17/regRNP17 (lane 2) results in a consistent increase in methylation of the C62 site, when normalized to an upper band which is not a methylation site and compared to that found in control. The right panel of Fig. 3B is an adjusted image of the left panel and more clearly shows the increase in U6 snRNA C62 methylation upon scaRNA17 reduction. Quantification of this and other experiments shows that scaRNA17 reduction significantly increases C62 methylation by 1.4-fold (Fig. 3C). RegRNP17, therefore, can impact the methylation of 18S rRNA at A484 and U6 snRNA at C62.

### Methylation of 28S rRNA G3923 is negatively regulated by regRNP2

**Fig. 4.**
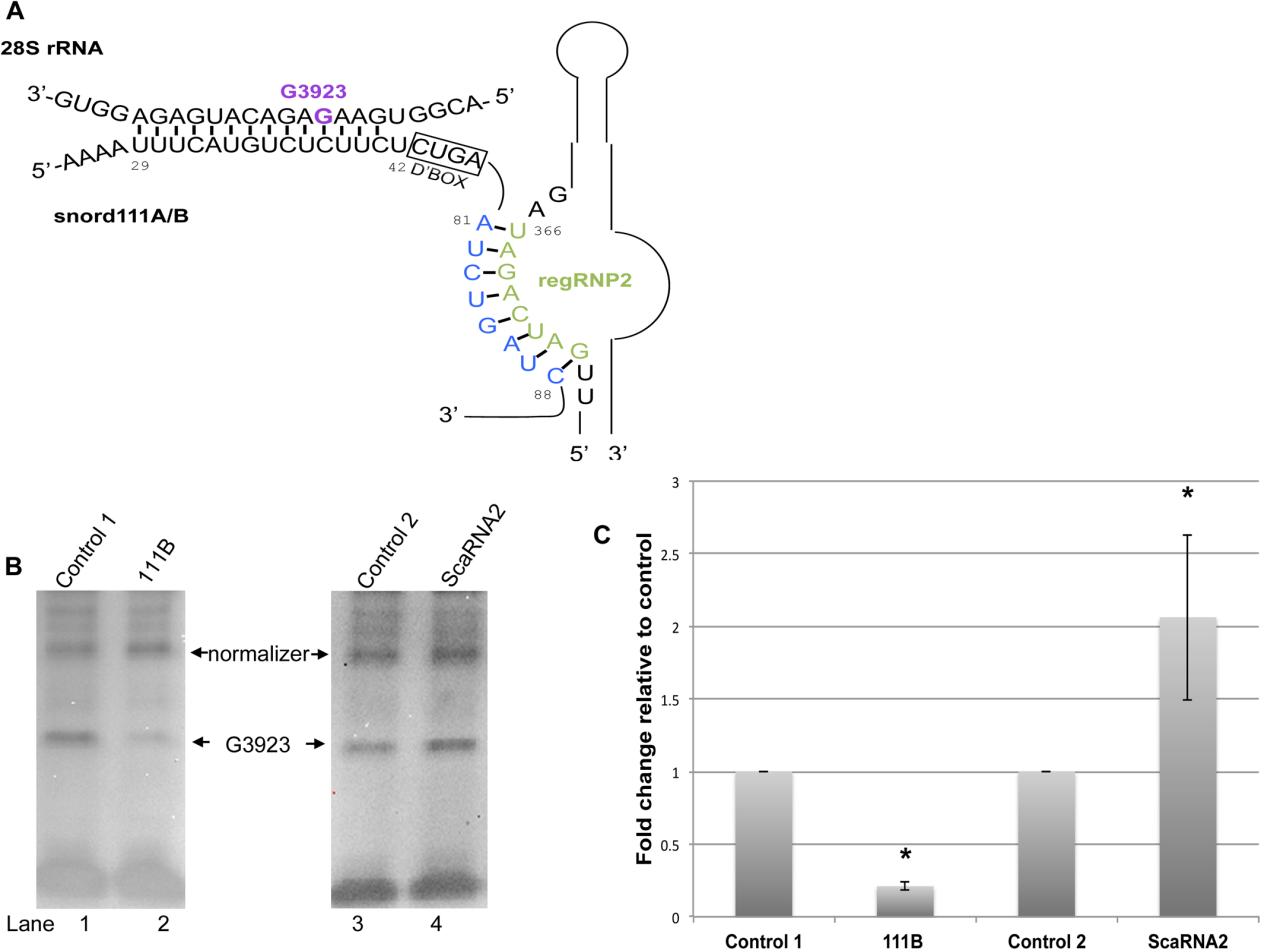
**Reduction of scaRNA2 increases 28S rRNA G3923 methylation.** (A) Complimentary alignment of snord111A/snord111B with 28S rRNA. Also shown is the base pairing of regRNP2 with snord111A/B. (B) RNA isolated from cells treated with control 1 or snord111B ASO (left panel), or control 2 or scaRNA2 siRNA (right panel), was subjected to primer extension with low (5 uM) dNTP levels. The location of the G3923 methylation stop is indicated, as is a band used for normalization. Quantification of this and other data are shown in C. Relative to control 1, reduction of the snord111B guide RNA decreases G3923 methylation by 71% (*n*=5, *P=0.002). A 2.1-fold increase in G3923 methylation is observed after scaRNA2 knockdown compared to control 2 (*n*=4, *P=0.002).

We next evaluated the potential of the nucleolus-enriched fragment derived from scaRNA2 [mgU2-61 (regRNP2) (Fig. 1A)] to affect RNA methylation. As shown in Fig. 4A, the antisense loop of regRNP2 can interact with snord111A or snord111B. Both snord111A and snord111B are predicted to guide the methylation of 28S rRNA G3923. To examine the impact of regRNP2 on the methylation of 28S rRNA G3923, scaRNA2 levels were reduced by RNAi followed by RNA isolation and primer extension analysis. We also examined the level of G3923 methylation after reduction of snord111B by ASO. These data are shown in Fig. 4B and, as expected, reduction of snord111B decreases the amount of 28S rRNA G3923 methylation compared to that obtained from cells treated with control ASO (left panel, compare the intensity of the G3923 band in lane 2 to that in lane 1). In these experiments snord111B was reduced approximately 70% as determined by qRT-PCR. Snord111A levels were not altered by the snord111B ASO. As discussed below, snord111B is approximately four times more abundant that snord111A. In opposition to that observed upon snord111B reduction, decreases in the amount of scaRNA2 increased the relative amount of G3923 methylation (Fig. 4, right panel, compare the amount of G3923 signal in lane 4 compared to that for the control siRNA reaction shown in lane 3). Quantification of these data was performed (Fig. 4C) using an upper band for normalizing and setting the control reactions to 100%, the results demonstrate that snord111B ASO treatment significantly reduces G3923 methylation by 71%. In contrast, reduction of scaRNA2 significantly increases G3923 methylation by 2.1-fold, strongly suggesting that regRNP2 is a negative regulator of 28S rRNA methylation at site 3923. For these experiments, scaRNA2 was typically reduced by 90%, as determined by qRT-PCR.

### Snord111B has regulatory RNP activities

**Fig. 5.**
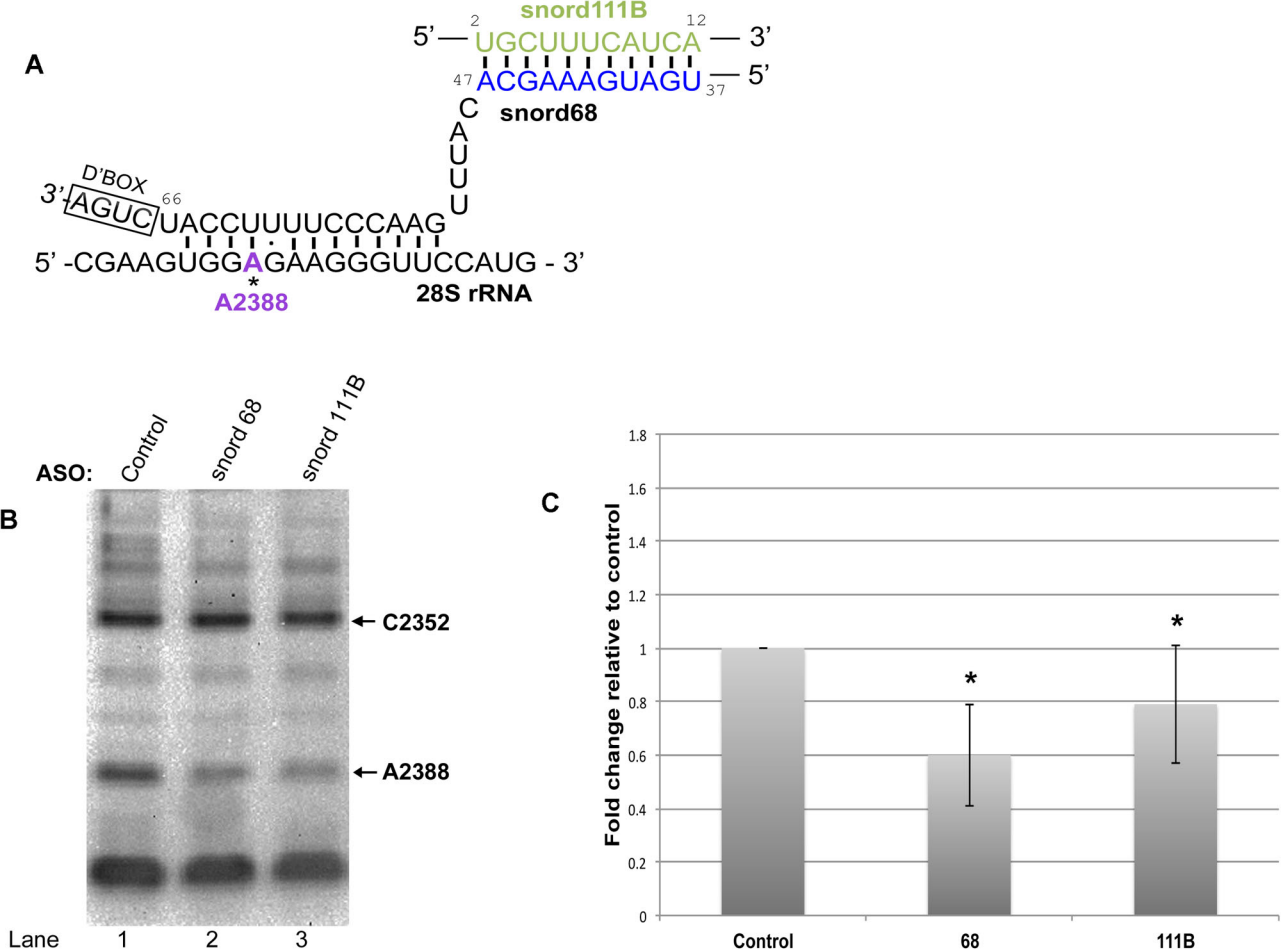
**Positive regulation of 28S rRNA A2388 methylation by snord111B.** (A) Schematic of the complimentary alignment of the snord68 guide RNA with 28S rRNA. Also shown is the base pairing between snord68 with snord111B. Note that the nucleotides of snord111B that can base pair with snord68 are absent in snord111A. (B) RNA was isolated from cells treated with control, snord68 or snord111B ASO and subjected to primer extension with low (2.5 µM) dNTP to interrogate 28S rRNA A2388 methylation. The C2352 methylation band was used as a normalizer. (C) Quantification of the data in B, along with other data. The relative amount of A2388 methylation in control RNA was set to 100%. A 40% decrease in A2388 methylation is observed when snord68 is reduced (*n*=10). A 21% decrease in A2388 methylation is seen when snord111B is reduced (*n*=9). Asterisks indicate *P*<0.05.

**Fig. 6.**
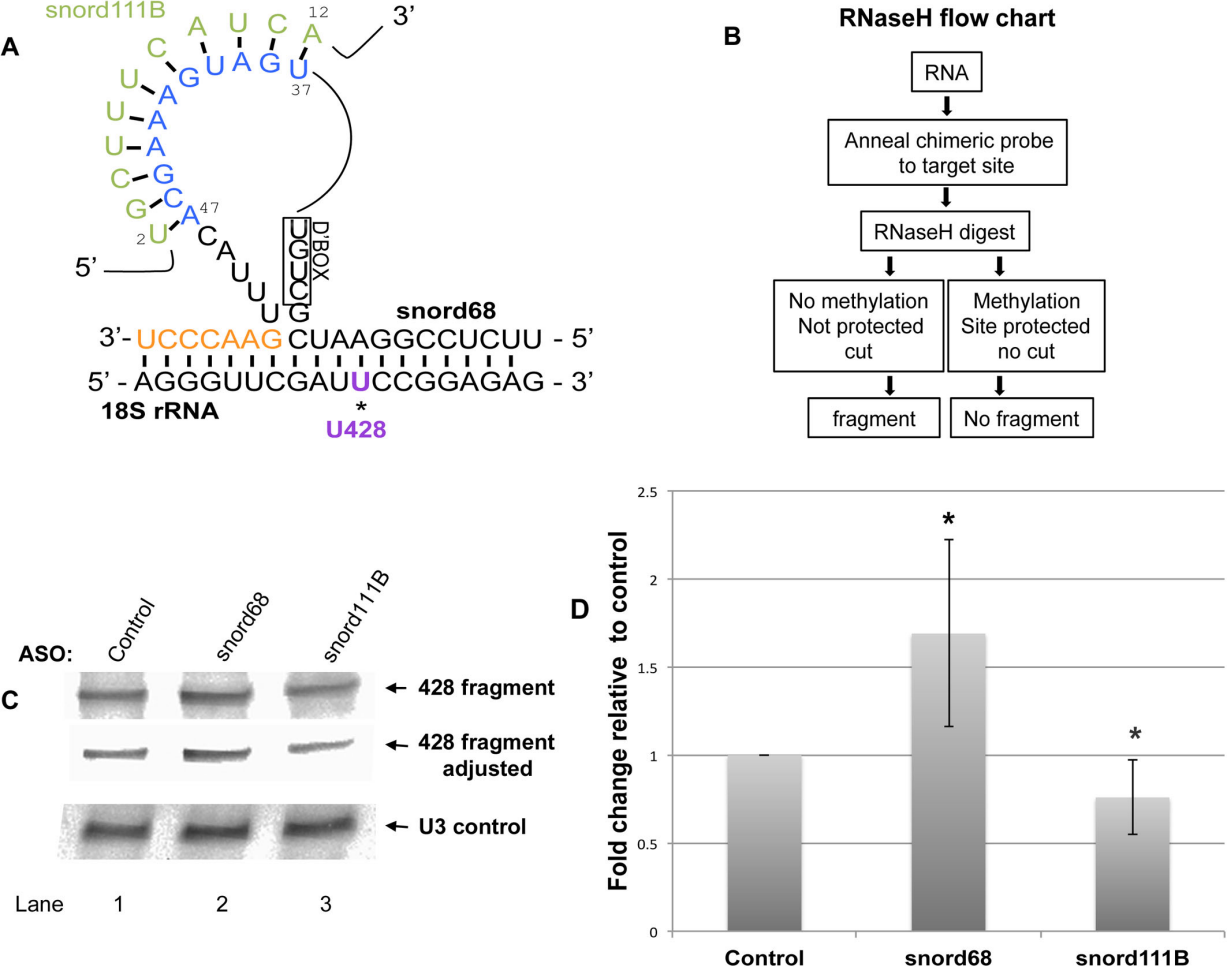
**Negative regulation of 18S rRNA U428 methylation by snord111B.** (A) Schematic of the complimentary base pairing of the snord68 guide RNA with 18S rRNA. The U428 methylation site is indicated. ‘Extra base pairings’ of snord68 with 18S rRNA (orange nucleotides) generates a loop in snord68. This loop region can base pair with snord111B. Note that the nucleotides of snord111B that interact with snord68 are not present in snord111A. (B) Flow chart for chimeric oligonucleotide-directed RNaseH cleavage assay to detect methylation. Methylated sites will be protected from RNaseH cleavage and will decrease the amount of 18S rRNA 428 fragment detected by northern blotting. (C) RNA isolated from cells transfected with control, snord68 or snord111B ASO was subjected to chimeric oligonucleotide-directed RNaseH cleavage to ascertain the amount of 18S rRNA U428 methylation. An adjusted image of the top panel is also shown to more easily visualize the differences between the reactions (middle panel). The blot was reprobed with a snord3 (U3) snoRNA probe to verify equal RNA loading in each reaction (bottom panel). (D) This and other data were quantified by normalizing the 428 fragment signal to the corresponding U3 snoRNA signal and setting the control ratio as 100%. Snord68 ASO treatment increases the amount of the 428 fragment by 1.7-fold compared to that obtained from control (*n*=6). Snord111B ASO treatment reduces the amount of the 428 fragment by 24% compared to control (*n*=10). Asterisks indicate *P*<0.05.

We next examined the regulation of snord68, which is responsible for the modification of a site within 28S rRNA (Fig. 5) and 18S rRNA (Fig. 6). As mentioned above, previous work has shown that many snoRNA guides form a loop region when interacting with target rRNA sites due to ‘extra base pairing’ (van Nues et al., 2011). The snoRNA loop forms between interaction sites of the snoRNA with rRNA. Such a loop is predicted for snord16 upon interaction with 18S rRNA and regRNP17 interacts with this loop region (Fig. 2A). Another example of a loop region formed by a guide RNA is seen for snord68 interaction with 18S rRNA (Fig. 6A, ‘extra base pairing’ indicated in orange). Snord68 guides the methylation of 18S at position U428. BLAST searches identified snord111B as a putative interactor of the loop region of snord68 (Fig. 6A). In addition to U428 of 18S rRNA, snord68 is also predicted to guide the methylation of 28S rRNA A2388 (Fig. 5A). The interaction of snord68 with 28S rRNA involves some of the same nucleotides that are used to form the ‘extra base pairing’ with 18S rRNA (compare Fig. 5A with Fig. 6A). Considering that snord111B aligns with the loop region of snord68, we next tested if snord111B may, like regRNP2 or regRNP17, have an impact on RNA methylation. Specifically, we examined the methylation of 28S rRNA site A2388 and 18S rRNA site U428 upon reduction of snord68 and snord111B. For these experiments, cells were treated with control, snord68, or snord111B ASOs. After 48 h of treatment, RNA was isolated and subjected to primer extension with low dNTPs levels to interrogate A2388 methylation in 28S rRNA (Fig. 5B). Compared to control ASO treated cells, RNA isolated from snord68 or snord111B ASO treated cells showed a reduction in the level of A2388 methylation (compare the intensity of the A2388 band in lanes 2 and 3 with that in lane 1). These data were quantified by normalizing the A2388 signal to the signal for another methylation site (at C2352) and normalizing this ratio to that observed with control ASO (Fig. 5C). As expected, reduction of the snord68 guide RNA decreased the relative level of A2388 methylation by 40%. Interestingly, snord111B reduction was also associated with a decrease in A2388 methylation, suggesting that snord111B is a positive regulator of 28S rRNA A2388 methylation. For these experiments, snord68 and snord111B were typically reduced by 60% and 70%, respectively, as determined by qRT-PCR.

We next examined the impact of snord111B on snord68-mediated methylation of 18S rRNA U428 (Fig. 6A). We were unable to design an effective primer to interrogate the methylation of U428 with low levels of dNTP in a primer extension assay. Consequently, we utilized another method to detect methylated sites which utilizes a chimeric oligonucleotide (consisting of DNA and 2′-*O*-methylated RNA) and RNaseH (Yu et al., 1997). As shown in the flow chart in Fig. 6B, the chimeric oligonucleotide is annealed to the target site of the RNA (U428 in this example). Subsequent digestion with RNaseH will be influenced by the methylation of U428. 18S rRNA that is not methylated at this site will not be protected by RNase H and hence will be cut. If U428 is methylated, however, the cut site is protected from RNase H and 18S rRNA is not cut. Reaction products are then resolved on a gel followed by northern blotting with a probe that detects the 18S rRNA fragment upstream of U428. The intensity of the fragment is thus inversely proportional to the amount of U428 methylation; more fragment means less methylation and less fragment means more methylation. RNA was isolated from cells treated with control, snord68 or snord111B ASOs for 48 h, followed by chimeric oligonucleotide directed RNase H cleavage assay to detect 18S rRNA U428 methylation. As shown in Fig. 6C, the amount of the U428 fragment is increased in snord68 ASO treated cells compared to that obtained with control ASO (compare the intensity of the band in lane 2 with that in lane 1). This finding is consistent with snord68 being the guide RNA for the methylation of U428. Specifically, reduction of snord68 guide RNA reduces U428 methylation which increases the amount of the fragment detected in the RNase H cleavage assay. In contrast, reduction of snord111B is associated with reduced U428 fragment levels when compared to control (compare intensity of band in lane 3 with that in lane 1). These data support the hypothesis that snord111B negatively regulates U428 methylation. To quantify this and other data, the U428 fragment signals were normalized to the signal obtained for snord3 (U3) detected on the same membrane (Fig. 6D). These data show that snord68 ASO treatment significantly reduces U428 methylation, as evidenced by a 1.7-fold increase in the amount of the U428 fragment. A small but significant decrease in the amount of U428 fragment detected in RNA isolated from cells with reduced levels of snord111B demonstrates that snord111B can serve as a regRNP that negatively regulates U428 methylation. Collectively, we provide evidence that snord111B can serve as a regulatory RNP that positively regulates the methylation of 28S rRNA A2388 (Fig. 5), but negatively regulates the methylation of 18S rRNA U428 (Fig. 6) in addition to its guide activity for 28S rRNA G3923 methylation (Fig. 4).

### Differential roles of snord111B and snord111A

**Fig. 7.**
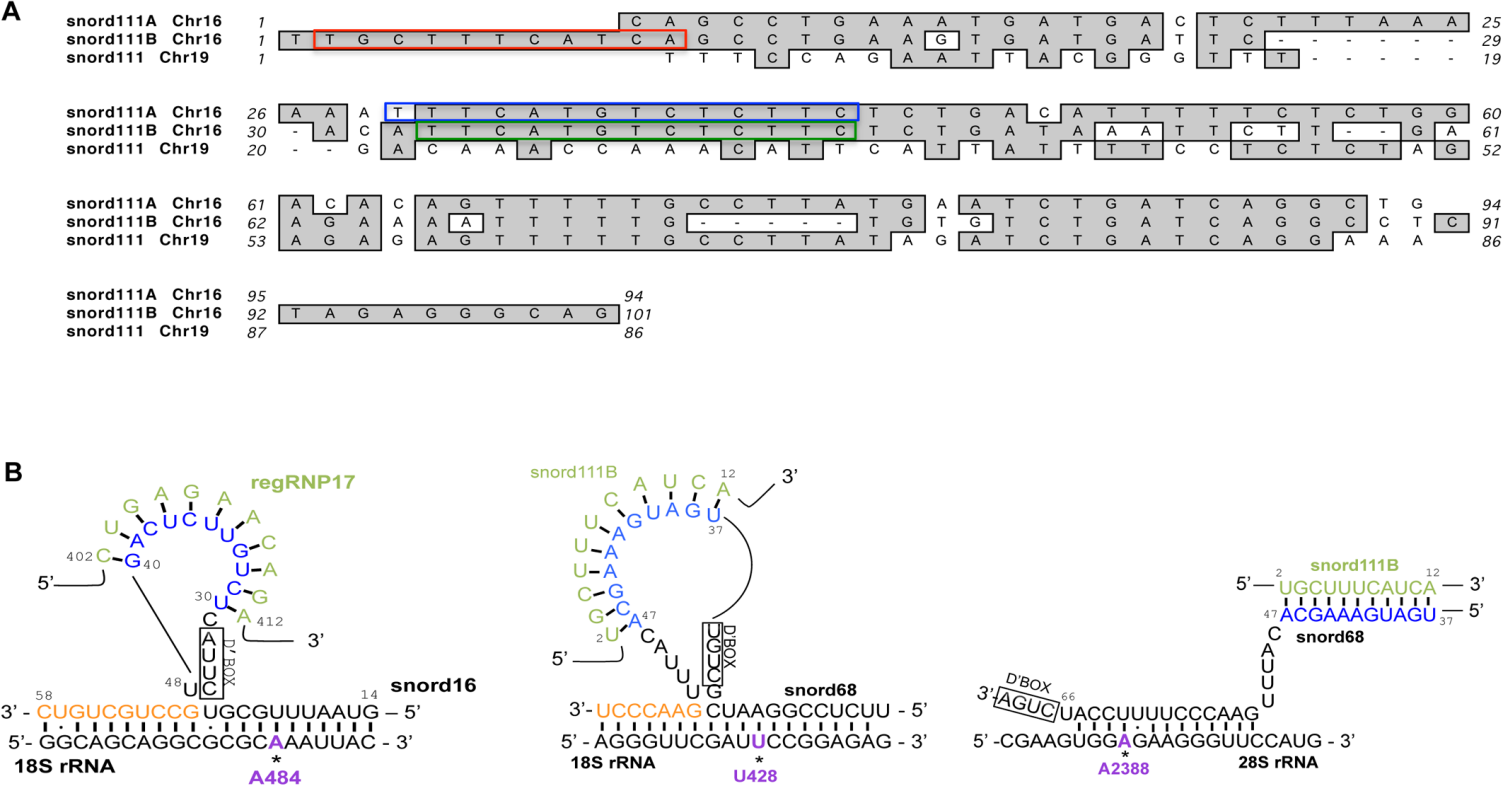
**Snord111 variants and snord111B interactions.** (A) Snord111A and snord111B are found on chromosome 16 while another snord111, of unknown function, is found on chromosome 19. Snord111A/B act as methylation guides for 3923 of 28S rRNA. The guide regions of 111A/B that base pair with 28S are highlighted in blue and green, respectively. The snord111B region that acts as a methylation regulator by base pairing with snord68, the mg snoRNA for both U428 of 18S RNA and A2388 of 28S rRNA, is highlighted in red. Snord111B and 111A are encoded in different introns of the host gene *SF3B3*. (B) Complimentary base pairing of regRNP17 with snord16 and snord111B with snord68. Note that the base pairing of snord111B with snord68 takes place via a loop of snord68 when snord68 interacts with 18S rRNA for U428 methylation. However, when snord68 interacts with 28S rRNA for A2388 methylation, the interaction of snord111B with snord68 may have a different confirmation. We have found that the loop regions formed by snord16 and snord68 are associated with negative regulation by regRNP17 and snord111B, respectively.

Snord111B and snord111A are both predicted to guide the methylation of 28S rRNA G3923, and the nucleotides that base pair with 28S rRNA are highly conserved (Fig. 7A, green and blue boxes). Both snord111B and snord111A are encoded within different introns of the *SF3B3* host gene on chromosome 16. Additionally, there is a snord111 related sequence on chromosome 19, but the region that interacts with 28S rRNA is not conserved in this variant (Fig. 7A). Although both snord111B and snord111A can guide the modification of G3923 in 28S rRNA, snord111B is unique in that nucleotides 2-12 (Fig. 7A, red box) can base pair with snord68 (Figs 5 and 6). Since snord111A does not contain a sequence that interacts with snord68, we believe that the altered methylation of 28S rRNA A2388 and 18S rRNA U428 shown in Figs 5 and 6 is imparted by snord111B. Analysis of snord111B and snord111A expression levels by qRT-PCR show that snord111B, despite being expressed from the same host gene, is approximately four times more abundant than snord111A.

As mentioned previously, many snoRNA guides are able to form a loop that allows for ‘extra base pairing’ with target RNA (van Nues et al., 2011). Both regRNP17 and snord111B can base pair with the loop regions formed by snord16 and snord68, respectively (Fig. 7B). Intriguingly, the association of regRNP17 and snord111B with these loop regions is correlated with negative regulation of the corresponding snoRNP. In contrast, snord111B interaction with snord68 in the context of 28S rRNA A2388 methylation is not proposed to take place within a similar loop region as found when snord68 interacts with the U428 target in 18S rRNA (Fig. 7B). Our methylation analysis indicates that, in the context of snord68 guiding 28S rRNA A2388 methylation, snord111B positively regulates snord68 activity. Thus, although 28S rRNA A2388 and 18S rRNA U428 methylation utilize the same snord68 and snord111B components, regulation of the methylation at these sites may differ in response to their local interaction context.

## DISCUSSION

The goal of the work presented here was to functionally demonstrate the existence of additional regulatory RNPs that could influence the methylation of RNA. We propose that regRNPs, via interactions with the snoRNA component of snoRNPs, can positively or negatively regulate the ribose methylation of rRNA and the nucleolar trafficked U6 snRNA. RegRNPs, therefore, may contribute to ribosome heterogeneity and spliceosomal function. When combined with our previously published results (Poole et al., 2017), we have now demonstrated that four methylation sites within rRNA and one methylation site within U6 snRNA is subjected to regRNP control (Table 1). This regulation can be positive or negative. Very significantly, we have shown that regRNP activity can be assigned to a snoRNP with a previously unclear function (snord111B) in addition to regRNPs derived from scaRNA 2 and 17. This finding strongly suggests that other snoRNPs besides snord111B may also possess regRNP activity. Indeed, there are many snoRNPs with no known function (Jorjani et al., 2016), so it is possible that these orphan snoRNPs contribute to ribosome heterogeneity by interacting with guide snoRNAs and affecting their activity.

**Table 1.**
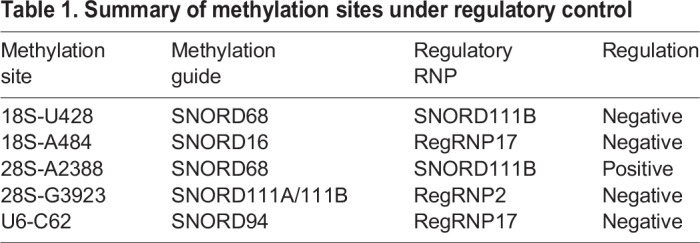
**Summary of methylation sites under regulatory control**

An important finding of our work is that the sites we have shown to be impacted by regRNPs are known to have variable levels of methylation (Table S1). The data in Table S1 was gathered from three studies (Incarnato et al., 2017; Krogh et al., 2016; Sharma et al., 2017) which analyzed rRNA methylation in four different cell lines. Since the variable methylation for these and other sites within rRNA are not correlated with altered levels of snoRNPs (Krogh et al., 2016), we propose that regRNPs are a contributor to ribosomal heterogeneity by influencing snoRNP activity.

One consideration for the regulation of snoRNP activity by regRNPs is the relative abundance of these factors. ScaRNAs are typically thought to be less abundant than snoRNAs (Darzacq et al., 2002). We examined the levels of several different scaRNAs and snoRNAs by qRT-PCR and found that there are indeed some snoRNAs that are highly abundant compared to some scaRNAs (Table S2). Generally speaking, scaRNA 2, 9 and 17 are more abundant than scaRNA 5 and 10, with scaRNA9 approximately 72% as abundant as GAPDH message. Note that for scaRNA 2, 9 and 17, primers were used that could amplify the processed fragments (regRNP2, regRNP9b and regRNP17) as well as the full-length scaRNAs. When considering the abundance of the snoRNAs with the cognate regRNPs we showed are functional in the data presented here (snord111B, regRNP2, regRNP17, Table 1), it is clear from the data in Table S2 that the snoRNA guide is more highly abundant. Thus, it is likely that regRNPs act catalytically and are not needed to be in a 1:1 stoichiometric ratio in order to have a functional consequence on snoRNP activity and ribose methylation. For example, the abundance of snord111B is 3.5 times less than that observed for snord68, but we detect a clear regulatory contribution for snord111B in the snord68-guided methylation of 18S rRNA U428 and 28S rRNA A2388. Collectively, our functional data shown in Figs 2–6 demonstrate that regRNPs (or snoRNPs that have regRNP activity) can impact ribose methylation of rRNA and the nucleolar trafficked U6 snRNA despite not being as abundant as the snoRNA guide RNAs they target.

In summary, the work we have described here further expands the known regRNPs that impact ribose methylation of rRNA and U6 snRNA. Specifically, we have now identified regRNPs that influence the modification of two sites within 18S rRNA (U428, A484), two sites within 28S rRNA (A2388, G3923) and one site within U6 snRNA (C62) (Figs 2–6, Table 1). Our current efforts seek to identify other regRNPs or snoRNPs that have regRNP activity. Additionally, the mechanisms by which regRNPs impart positive or negative regulation of snoRNP activity is also an active area of current research. For example, we have shown by RNase protection assays using RNA fragments that binding of regRNP17 to snord16 disrupts the interaction of snord16 with 18S rRNA (Poole et al., 2017). It is possible that other regRNPs that act in a negative manner utilize a similar strategy to disrupt guide snoRNA base pairing to target rRNA. Conversely, regRNPs that act in a positive manner may stabilize snoRNA/rRNA interactions in much the same way that snRNA–snRNA interactions facilitate spliceosomal function. These and other experimental approaches will further clarify how rRNA methylation contributes to ribosome heterogeneity. Such information will likely become more important as an assessment of rRNA methylation in various diseases becomes more standard. Ribosome specialization (due in large part to alterations in rRNA methylation) has already been shown to contribute to tumorigenesis (Marcel et al., 2015, 2013; Truitt and Ruggero, 2016). Other work has shown that increased levels of 28S rRNA methylation is associated with β-thalassemia trait carriers (Sornjai et al., 2017). The identification of additional regRNPs that impact rRNA methylation and a characterization as to how these complexes function is, therefore, timely and very significant.

## MATERIALS AND METHODS

### Cell lines, cell culture, and transfections

HeLa cells were obtained from the American Type Culture Collection (Manassas, USA) and were cultured in DMEM media (Invitrogen) supplemented with 10% heat inactivated fetal bovine serum (Gibco) and 1% penicillin streptomycin (Corning, Manassas, USA). Cells were cultured in a 5% CO_2_ incubator at 37°C.

DsiRNA and antisense oligonucleotide (ASO) transfections were performed using Lipofectamine RNAiMax (Invitrogen) according to the manufacturer's suggested protocol with 48 h incubation. The following dsiRNAs were obtained from Integrated DNA Technologies (Coralville, USA): scaRNA2 sense (5′-rUrGrArUrUrArUrCrGrArGrGrCrGrArUrUrCrUrGrArUrCTG-3′), anti-sense (5′-rCrArGrArUrCrArGrArArUrCrGrCrCrUrCrGrArUrArArUrCrArGrU-3′), scaRNA17 sense (5′-rCrCrGrCrArGrUrArUrUrUrUrCrCrUrUrArUrArUrGrArUCA-3′), anti-sense (5′-rUrGrArUrCrArUrArUrArArGrGrArArArArUrArCrUrGrCrGrGrGrC-3′). ASOs were also obtained from Integrated DNA Technologies, and are as follows: snord16 sense control (5′-mU*mC*mA*mG*mC*G*A*C*A*G*T*T*G*C*C*mU*mG*mC*mU*mG-3′), snord16 antisense (5′- mC*mA*mG*mC*mA*G*G*C*A*A*C*T*G*T*C*mG*mC*mU*mG*mA -3′), scaRNA17 (5′- mA*mU*mA*mU*mA*A*G*G*A*A*A*A*T*A*C*mU*mG*mC*mG*mG -3′), snord68 antisense (5′-mA*mU*mG*mU*mG*C*T*T*T*C*A*T*C*A*A*mG*mG*mC*mC*mG-3′), snord111B antisense (5′-mG*mA*mA*mG*mA*G*A*C*A*T*G*A*A*T*C*mU*mG*mA*mA*mU-3′). Note, the m denotes 2′ *O*-methylated RNA bases and the * denotes phosphorothioate bonds.

### Quantitative real-time PCR

RNA was extracted from 48 h transfected HeLa cells with TRI-REAGENT (Cincinnati, USA) according to the manufacturer's suggested protocol. Reactions were set up with 50 ng total RNA in Brilliant II SYBR Green qRT-PCR master mix (Agilent, Santa Clara, USA) using an Agilent MX3000P qRT-PCR system. Amplification rates, Ct values and dissociation curve analyses of products were determined using MxPro (version 4.01) software. Relative expression was determined using the 2^-ΔΔCT^ method (Livak and Schmittgen, 2001). Microsoft Excel was used for post-hoc statistical analysis using the Student's *t*-test. Oligonucleotides used were obtained from Integrated DNA Technologies and were as follows: GAPDH forward (5′-GACTCATGACCACAGTCCATGCCATC-3′), reverse (5′-GACTCATGACCACAGTCCATGCCATC-3′), scaRNA2 forward (5′-CGTGTTAGGCGAGTGCGTGCGCCCACC-3′), reverse (5′-CTCGCTGCACAGCGCCCCGAGGGGGC -3′), snord111A, forward (5′-CAGCCTGAAATGATGACTC-3′), reverse (5′-CAGCCTGATCAGATTCATAAGG-3′), snord111B forward (5′-TGTTTTCATCAGCCTGAAGTG-3′), reverse (5′-GAGGCCTGATCAGACACACA-3′), snord68, forward (5′-CGTGATGACATTCTCCGGAATC-3′), reverse (5′-AAATGTGCTTTCATCAAGGCCG-3′), scaRNA17 forward (5′- GCTGGACCCGGACCGGTTTTGGG-3′), reverse (5′-AAGGAAAATACTGCGGGCTCATCC-3′), sca2 forward (5′- GAAGTGATGAATTGATCAGATAGACG-3′), reverse (5′-ATCAGAATCGCCTCGATAATCA-3′), sca17 forward (5′- GGCCGATGATGACGAGACCACTG-3′), reverse (5′-CGGCCTCAGTCTGTTCTCAGAAC-3′), scaRNA9 forward (5′- GGGCAATGATGAAAAGGTTTTACTACTGATCTTTG-3′), reverse (5′-TGAGCTCAGGTCAAGTGTAGAAACCATC-3′), scaRNA10 forward (5′- GCCACATGATGATATCAAGGCTG-3′), reverse (5′-GCCATCAGATTACCAAAGATCTGTG-3′), scaRNA5 forward (5′- GGTCGATGATGATTGGTAAAAGGTC-3′), reverse (5′-GGTCTCAGATTGAAAACTTGAGATC-3′), snord45A forward (5′- ATCGAGGCTAGAGTCACGCTTG-3′), reverse (5′-GCATGTCTCTAACCTGGTGAC-3′), snora43 forward (5′- GCTGTCCTGGACCTGTTGGCACC-3′), reverse (5′-GTCAGGCCATAAACCATTCTC-3′), snord16 forward (5′- TGCAATGATGTCGTAATTTGCG-3′), reverse (5′-TTGCTCAGTAAGAATTTTCGTC-3′), snord100 forward (5′- TGACAACTGGCTCCCTCTACT-3′), reverse (5′-GGTGACATGGCAGTTTCCTC-3′), snord15A forward (5′- CTTCGATGAAGAGATGATGAC-3′), reverse (5′-CCTTCTCAGACAAATGCCTC-3′), snord94 forward (5′- CAGGCTGTGATGATTGGCGCAG-3′), reverse (5′-CAGGCTCAGATTGAGGCAACAG-3′), snora70A forward (5′- CATGGGGACCCAGTGTGCG-3′), reverse (5′-CATACAACCAACAGGCTGCG-3′).

### Primer extension assay to detect 2′-O-methylation of RNA

RNA was extracted from 48 h transfected HeLa cells with TRI-REAGENT according to the manufacturer's suggested protocol. 2 µg RNA was prepared with 1 µl Reverse Transcriptase buffer (New England Biolabs, Ipswich, USA), 1 µl of 5 µM dig labeled primer designed to base pair downstream of the methylation site of interest (Integrated DNA Technologies) and DEPC H_2_O to 8 µl. After 2 min at 95°C and 10 min at 42°C, 1 µl Reverse Transcriptase (New England Biolabs) and 1 µl dNTPs were added and samples returned to 37°C for 1 h. The amount of dNTPs used are as noted, or were low concentrations (2.5 µM or 5 µM) used to detect ribose methylation. Samples plus loading buffer were run on a pre-warmed 15% TBE urea gel (Invitrogen) in 1X TBE at 180 volts for 80 min. Gel was then rinsed in 1X TBE for 10 min. cDNA product was transferred to membrane using iBlot DNA transfer stacks (Invitrogen) with the iBlot Gel Transfer device (Life Technologies) using program 5 for 3 min, rinsed in ultrapure H_2_0 and crosslinked at 120 K uJ/cm2. Membrane was incubated in Roche 1X blocking buffer for 15 min with slow rotation, then 30 min with slow rotation in Roche Anti-Digoxigenin-AP Fab fragments at 1:10,000 in Roche blocking buffer and washed with slow rotation in 1X Wash Buffer, [Roche wash and block buffer set, (Roche, Mannheim, Germany)]. Membrane was developed with 1X CSPD in development buffer at 1:100, 5 min at room temp, then placed between transparencies for 15 min in a 37°C incubator. Chemiluminescent images were captured with a BIORAD Chemi Doc Universal Hood and Quantity One Software (Bio-Rad). Digoxigenin labeled DNA oligonucleotides were obtained from Integrated DNA Technologies and are as follows: 18S rRNA A484 site; 5′-DiGN/GCGCGCCTGCTGCCTTCCTTGGA-3′, 28S rRNA G3923 site; 5′-DiGN/CGCCGGGGGCCTCCCACTTATT-3′, U6 C62 site; 5′-DiGN/ACGCTTCACGAATTTGCGTG-3′, 28S rRNA A2388 site; 5′-DiGN/CCCATGTTCAACTGCTGTTCAC-3′.

### RNAseH cleavage assay

RNA was extracted from HeLa cells with TRI-REAGENT according the manufacturer's suggested protocol. 1.5 µg RNA in 4 µl H_2_0 was combined with 1 µl of 100 ng/µl chimeric probe designed to base pair with the RNA at the methylation site of interest. Samples were heated at 90°C for 5 min and then 37°C for 10 min to allow annealing, followed by 2 min on ice. 1 µl 10X RNAseH buffer, 1 µl RNAseH, 1 µl of RNAse Inhibitor (all New England Biolab) and 2 µl H2O were combined and added to each sample and returned to 37°C for 60 min. For a marker, we used 10 µl of Dig III marker (Roche). 10 µl of Gel Loading Buffer II (Invitrogen) was added to each sample and marker. Samples were heated at 95°C for 5 min, loaded into a pre-run 6% urea gel (Invitrogen) in 1X TBE and run at 200V for 90 min. Transfer was performed using iBlot DNA transfer stacks (Invitrogen), rinsed briefly in ultrapure H_2_0, dried and crosslinked at 120 K uJ/cm2. Membrane was blocked, incubated with anti-dig and washed with slow rotation (Roche wash and block buffer set, and Roche anti-dig at 1:10,000). Membrane was developed with 1X CSPD in development buffer at 1:100, 5 min at room temp followed by 15 min in a 37°C incubator. Chemiluminescent images were captured with a BIORAD Chemi Doc Universal Hood and Quantity One Software.

Chimeric oligonucleotide was obtained from Integrated DNA Technologies as follows: 18S U428 site, 5′-mGmGAATCmGmAmAmCmCmCmUmGmAmUmUmC-3′ (the m denotes 2′ *O*-methylated RNA bases). The Digoxigenin labeled probe to anneal to the 5′ end of 18S RNA upstream of U428: /5DiGN/GACAAGCATATGCTACTGGCAGGATCAACCAGGTA.

### Blast searches and Genome browsing

Blast searches for possible RNA interactions with snoRNAs were performed using publicly available databases (Jorjani et al., 2016; Lestrade and Weber, 2006). For identification of snord coding sequences in the genome, we utilized the University of California, Santa Cruz (UCSC) genome browser (Kent et al., 2002).

### Statistical analysis

Student's *t*-test was performed to determine statistical significance. Asterisk * indicates *P*≤0.05.

## Supplementary Material

Supplementary information

## References

[BIO036095C1] Belin S., Beghin A., Solano-Gonzàlez E., Bezin L., Brunet-Manquat S., Textoris J., Prats A.-C., Mertani H. C., Dumontet C. and Diaz J.-J. (2009). Dysregulation of ribosome biogenesis and translational capacity is associated with tumor progression of human breast cancer cells. *PLoS ONE* 4, e7147 10.1371/journal.pone.0007147 19779612 PMC2744998

[BIO036095C2] Darzacq X., Jady B. E., Verheggen C., Kiss A. M., Bertrand E. and Kiss T. (2002). Cajal body-specific small nuclear RNAs: a novel class of 2′-O-methylation and pseudouridylation guide RNAs. *EMBO J.* 21, 2746-2756. 10.1093/emboj/21.11.2746 12032087 PMC126017

[BIO036095C3] Incarnato D., Anselmi F., Morandi E., Neri F., Maldotti M., Rapelli S., Parlato C., Basile G. and Oliviero S. (2017). High-throughput single-base resolution mapping of RNA 2-O-methylated residues. *Nucleic Acids Res.* 45, 1433-1441. 10.1093/nar/gkw810 28180324 PMC5388417

[BIO036095C4] Jorjani H., Kehr S., Jedlinski D. J., Gumienny R., Hertel J., Stadler P. F., Zavolan M. and Gruber A. R. (2016). An updated human snoRNAome. *Nucleic Acids Res.* 44, 5068-5082. 10.1093/nar/gkw386 27174936 PMC4914119

[BIO036095C5] Kent W. J., Sugnet C. W., Furey T. S., Roskin K. M., Pringle T. H., Zahler A. M. and Haussler D. (2002). The human genome browser at UCSC. *Genome Res.* 12, 996-1006. 10.1101/gr.229102 12045153 PMC186604

[BIO036095C6] Kiss T. (2004). Biogenesis of small nuclear RNPs. *J. Cell Sci.* 117, 5949-5951. 10.1242/jcs.01487 15564372

[BIO036095C7] Krogh N., Jansson M. D., Häfner S. J., Tehler D., Birkedal U., Christensen-Dalsgaard M., Lund A. H. and Nielsen H. (2016). Profiling of 2′-O-Me in human rRNA reveals a subset of fractionally modified positions and provides evidence for ribosome heterogeneity. *Nucleic Acids Res.* 44, 7884-7895. 10.1093/nar/gkw482 27257078 PMC5027482

[BIO036095C8] Lafontaine D. L. J. (2015). Noncoding RNAs in eukaryotic ribosome biogenesis and function. *Nat. Struct. Mol. Biol.* 22, 11-19. 10.1038/nsmb.2939 25565028

[BIO036095C9] Lestrade L. and Weber M. J. (2006). snoRNA-LBME-db, a comprehensive database of human H/ACA and C/D box snoRNAs. *Nucleic Acids Res.* 34, D158-D162. 10.1093/nar/gkj002 16381836 PMC1347365

[BIO036095C10] Livak K. J. and Schmittgen T. D. (2001). Analysis of relative gene expression data using real-time quantitative PCR and the 2(-Delta Delta C(T)) method. *Methods* 25, 402-408. 10.1006/meth.2001.1262 11846609

[BIO036095C11] Maden B. E. H., Corbett M. E., Heeney P. A., Pugh K. and Ajuh P. M. (1995). Classical and novel approaches to the detection and localization of the numerous modified nucleotides in eukaryotic ribosomal RNA. *Biochimie* 77, 22-29. 10.1016/0300-9084(96)88100-4 7599273

[BIO036095C12] Marcel V., Ghayad S. E., Belin S., Therizols G., Morel A.-P., Solano-Gonzàlez E., Vendrell J. A., Hacot S., Mertani H. C., Albaret M. A. et al. (2013). p53 acts as a safeguard of translational control by regulating fibrillarin and rRNA methylation in cancer. *Cancer Cell* 24, 318-330. 10.1016/j.ccr.2013.08.013 24029231 PMC7106277

[BIO036095C13] Marcel V., Catez F. and Diaz J.-J. (2015). Ribosome heterogeneity in tumorigenesis: the rRNA point of view. *Mol. Cell Oncol.* 2, e983755 10.4161/23723556.2014.983755 27305893 PMC4905297

[BIO036095C14] Massenet S., Bertrand E. and Verheggen C. (2017). Assembly and trafficking of box C/D and H/ACA snoRNPs. *RNA Biol.* 14, 680-692. 10.1080/15476286.2016.1243646 27715451 PMC5519232

[BIO036095C15] Poole A. R., Vicino I., Adachi H., Yu Y.-T. and Hebert M. D. (2017). Regulatory RNPs: a novel class of ribonucleoproteins that potentially contribute to ribosome heterogeneity. *Biol. Open* 6, 1342-1354. 10.1242/bio.028092 28808137 PMC5612246

[BIO036095C16] Sharma S., Marchand V., Motorin Y. and Lafontaine D. L. J. (2017). Identification of sites of 2′-O-methylation vulnerability in human ribosomal RNAs by systematic mapping. *Sci. Rep.* 7, 11490 10.1038/s41598-017-09734-9 28904332 PMC5597630

[BIO036095C17] Sornjai W., Lithanatudom P., Erales J., Joly P., Francina A., Hacot S., Fucharoen S., Svasti S., Diaz J. J., Mertani H. C. et al. (2017). Hypermethylation of 28S ribosomal RNA in beta-thalassemia trait carriers. *Int. J. Biol. Macromol.* 94, 728-734. 10.1016/j.ijbiomac.2016.10.039 27765567

[BIO036095C18] Truitt M. L. and Ruggero D. (2016). New frontiers in translational control of the cancer genome. *Nat. Rev. Cancer* 16, 288-304. 10.1038/nrc.2016.27 27112207 PMC5491099

[BIO036095C19] Tycowski K. T., Aab A. and Steitz J. A. (2004). Guide RNAs with 5′ caps and novel box C/D snoRNA-like domains for modification of snRNAs in metazoa. *Curr. Biol.* 14, 1985-1995. 10.1016/j.cub.2004.11.003 15556860

[BIO036095C20] van Nues R. W., Granneman S., Kudla G., Sloan K. E., Chicken M., Tollervey D. and Watkins N. J. (2011). Box C/D snoRNP catalysed methylation is aided by additional pre-rRNA base-pairing. *EMBO J.* 30, 2420-2430. 10.1038/emboj.2011.148 21556049 PMC3116282

[BIO036095C21] Yu Y. T., Shu M. D. and Steitz J. A. (1997). A new method for detecting sites of 2′-O-methylation in RNA molecules. *RNA* 3, 324-331. 9056769 PMC1369484

