# Supplementary material for: Identification of additional regulatory RNPs that impact rRNA and U6 snRNA methylation
Source: Biol Open. 2018 Jul 23;7(8):bio036095. doi: 10.1242/bio.036095 (PMC6124571; doi:10.1242/bio.036095)
Supplement: Supplementary information [file biolopen-7-036095-s1.pdf]

Biology Open (2018): doi:10.1242/bio.036095: Supplementary information

Table S1. Methylation frequency from various studies for the sites investigated in this work.

	Sharma et al 2017 HCT116 p53+/+	Sharma et al 2017 HCT116 p53-/-	Krogh et al 2016 HCT116	Krogh et al 2016 HeLa	Incarnato et al 2016 HeLa S3
**18S - U428**	0.8	0.72	0.85	0.88	not reported
**18S - A484**	0.73	0.62	0.98	0.98	0.76 *
**28S - A2388**	0.69	0.54	0.83	0.71	not reported
**28S - G3923**	0.7	0.57	0.8	0.88	0.003*

* Methylation expressed as 2OMe Ratio

Table S2. Abundance levels (expressed as percent relative to GAPDH) of selected scaRNAs and snoRNAs.

RNA	% rel to GAPDH
scaRNA2	7.3
scaRNA9	72
scaRNA17	26
scaRNA5	2.7
scaRNA10	3.2
snord15A	276
snord16	409
snord45A	814
snord68	431
snord94	113
snord100	382
snord111A	28
snord111B	124
snora43	793
snora70A	89
